# A review of electroacupuncture in bone repair: Mechanisms and clinical implications

**DOI:** 10.1097/MD.0000000000040725

**Published:** 2024-11-22

**Authors:** Yu-Jun Gao, Yin-cang Wang, De-lai Zhao, Qing Wen, Hao-Xin Shi, Shu-Ren Wang

**Affiliations:** a Graduate School, Heilongjiang University of Chinese Medicine, Haerbin, China; b First Affiliated Hospital, Heilongjiang University of Chinese Medicine, Haerbin, China; c The Fifth Hospital of Harbin City, Haerbin, China.

**Keywords:** electroacupuncture, fracture healing, inflammation, neovascularization, pain

## Abstract

The journey of bone repair is a lengthy process. Traditionally, oral or topical medications have been employed to facilitate healing, approaches that are not only costly but may also lead to adverse effects such as gastrointestinal damage. With advancements in electrophysiology, the significance of bioelectric activity in tissue repair has become increasingly prominent, thereby enhancing the focus on research into electroacupuncture (EA) for bone repair. EA, a synthesis of traditional acupuncture and electrical stimulation, can regulate pain by inhibiting the transmission of electrical signals, reducing the expression of ion channel proteins, and promoting the release of neurotransmitters at targeted sites. Moreover, EA has the capability to influence macrophage polarization and modulate inflammatory cytokines, aiding in bone repair. Additionally, EA has the potential to regulate cytokines such as Ephrin type-B receptor 4 (EphB4), Vascular Endothelial Growth Factor (VEGF), Erythropoietin (EPO), and Bone Morphogenetic Protein 2 (BMP-2), thus promoting angiogenesis and fracture healing.This paper explores the mechanisms by which EA facilitates bone healing and assesses its advantages and limitations in clinical applications. It offers a theoretical foundation for the safe, effective, and rational use of EA, presenting a novel approach for enhancing bone regeneration.

## 1. Introduction

Fractures are a prevalent form of traumatic injury that significantly affect an individual’s heath and quality of life due to potential functional limitations or disability. Factors such as an aging population, increased use of non-steroidal anti-inflammatory drugs, smoking, and the rising prevalence of various illnesses contribute to the growing incidence of fracture-related complications such as infections and delayed healing. Globally, approximately 160 to 190 million new fractures occur each year. Additionally, over 400 million individuals suffer from the acute or long-term consequences of fractures. The incidence of fractures is notably high among those over the age of 18 and continues to rise with advancing age.^[1]^ Fractures can lead to persistent pain, functional disability, and increase the economic burden on families and society^[2–4]^, This also amplifies the psychological stress experienced by the patients.

Fracture healing is a complex and lengthy process that, although initiated at the time of the fracture, still requires 8 to 16 weeks to reach the standard of clinical healing, and complete osseous healing takes even longer.^[5]^ In the early stages of a fracture, the initial bleeding and hematoma formation lay the foundation for repair. Neutrophils arrive first to clear the damaged area, followed by monocytes differentiating into macrophages, releasing inflammatory factors to recruit repair cells. Subsequently, fibroblasts and chondroblasts secrete matrix in a low-oxygen environment to form a cartilage scaffold. With the restoration of blood supply, osteoblasts replace chondroblasts to produce new bone. In the remodeling phase, osteoclasts and osteoblasts work together to regulate bone structure to meet mechanical demands, with osteoblasts strengthening and rebuilding bone tissue.^[6]^ Currently, the recognized treatment concepts after a fracture are reposition, fixation, and rehabilitation exercise, and although the methods of traditional Chinese medicine and Western medicine differ, they generally follow the same concept. However, in the field of promoting bone repair, many treatment options such as growth factor therapy,^[7]^ stem cell therapy,^[8]^ and platelet-rich plasma (PRP) have emerged.^[9]^ While these therapies have certain therapeutic potential, they also come with significant side effects. For example, growth factor therapy requires precise control over the delivery of growth factors, but their short half-life in the body makes it difficult to maintain effective concentrations in target tissues, leading to potential off-target effects; stem cell therapy may have the risk of tumorigenesis; platelet-rich plasma therapy may lead to local inflammation and infection risks. Furthermore, these therapies often incur high costs, significantly increasing medical expenses and financial burden. Therefore, there is a need for an effective, low-side-effect, and cost-efficient treatment method to replace existing treatments to promote bone repair, avoid delayed healing and other fracture complications, and reduce medical costs.

Electroacupuncture (EA), an extension of traditional Chinese acupuncture therapy,^[10]^ achieves therapeutic outcomes by applying electrical stimulation to specific points on the human body, Currently, In randomized controlled trials in clinical studies, EA showed significant efficacy in shortening clinical healing time and reducing the incidence of delayed healing.^[11,12]^ Furthermore, EA has shown positive effects in pain management^[13]^ and inflammation control.^[14]^ These research results highlight the significant potential of EA in promoting healing and have sparked exploration into the mechanisms of EA. In recent years, certain mechanisms of EA have been gradually elucidated, such as its effects on nerve conduction, macrophage polarization types, stem cell differentiation, and involvement in regulating the production and release of cytokines, such as serotonin (5-HT),^[15]^ interferon-gamma,^[16]^ and Vascular Endothelial Growth Factor (VEGF).^[17]^As an adjunct therapy, EA offers numerous advantages in the field of bone repair, being safe and effective without causing gastrointestinal damage, making it an excellent noninvasive anti-inflammatory, analgesic, and healing-promoting treatment method. Nevertheless, compared to Western medical treatments, data from large-scale clinical trials on EA are relatively scarce, and its effectiveness may vary due to individual patient differences (such as age, gender, and condition), with some patients potentially less responsive to EA therapy, potentially affecting its acceptance in certain medical settings. Therefore, further research is crucial to clarify the mechanism of EA in bone repair and to select the optimal timing and parameters for use.

In this review, we conducted an in-depth analysis of the role of EA in pain control, inflammation management, and tissue repair during the initial stages of fracture healing. We explored the application of EA in early fracture management, highlighting its biological foundations, clinical outcomes, and therapeutic benefits.

## 2. Analgesic effects of EA

**Figure 1.**
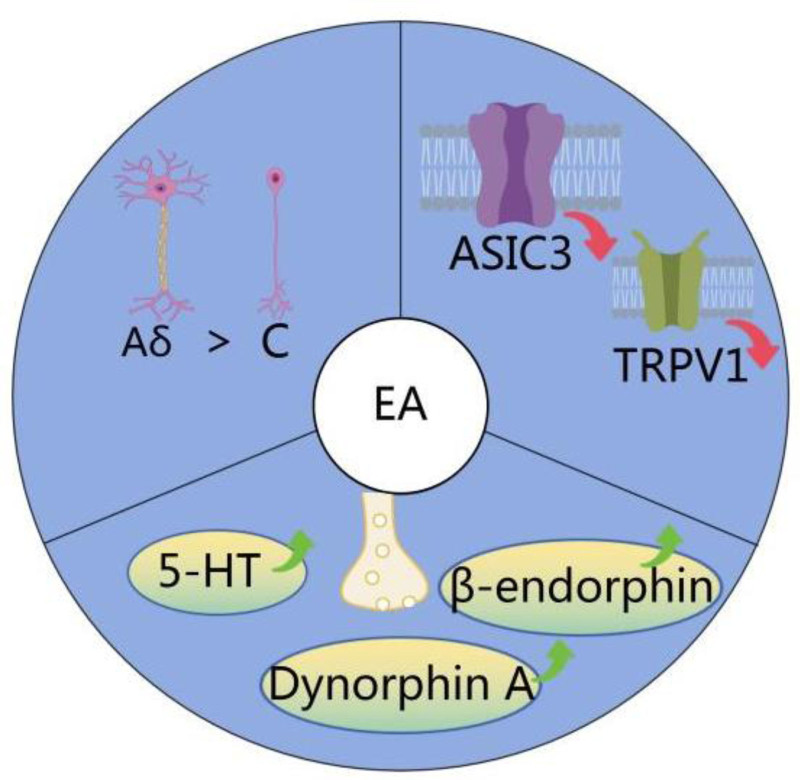
Mechanisms of EA analgesia. EA = electroacupuncture.

In the process of fracture healing, pain is an important cause of discomfort, often stems primarily from factors such as fractures, muscle strain, soft tissue damage, compression caused by hematomas, and inflammatory reactions.^[18]^ EA exerts its analgesic effects through various mechanisms, including neurobiological pathways, suppression of ion channel expression, and modulation of neurotransmitters (Fig. 1).

### 2.1. Neurobiological mechanism

Pain arising from fractures is predominantly due to pressure changes resultant from relative movement at the fracture site. This mechanically induced stimulation activates nerve fibers present in the periosteum attached to the bone surface. These nerve fibers are inherently sensitive to pressure and promote the rapid start of electrical signals.^[19]^These signals travel through the neurons’ axons, undergo initial processing in the dorsal horn of the spinal cord, and eventually reach the brain, culminating in the sensation of pain.^[20]^ Within the nervous system, sensory nerve fibers are classified into large-diameter Aβ fibers and small-diameter C fibers; Aβ fibers mainly relay touch and pressure signals while C fibers carry pain and temperature signals. The conduction velocity of electrical signals in Aβ fibers is greater than that in C fibers.^[21]^ In the dorsal horn of the spinal cord, a “gating” mechanism is present: stimulation of Aβ fibers activates inhibitory neurons that suppress the pain signals from C fibers. This mechanism acts as a gate that only opens under particular conditions, such as heightened activity of small fibers, permitting the passage of pain signals. Utilizing the differing conduction speeds of these nerve fibers can help block these electrical signals, thereby mitigating pain. EA employs this principle to inhibit the pain signals transmitted by small-diameter C fibers by activating large-diameter Aβ fibers, thus reducing pain perception. Specifically, EA stimulates inhibitory neurons in the dorsal horn, reducing the response to pain signals and effectively diminishing pain sensation.^[22,23]^

### 2.2. Suppress ion channel expression

The occurrence of pain in the early stage of fracture is related to the increased expression of various ion channels. These changes occur both in the central nervous system and in the periphery of sensory neurons, highlighting the important role of ion channels in pain management.^[24,25]^ Following a fracture, the breakdown of hemoglobin, engagement in anaerobic metabolism, and tissue necrosis precipitate a significant accumulation of acidic substrates.^[26]^ Acid-Sensing Ion Channel three (ASIC3), which has a prime function in detecting shifts in extracellular acidity, is vital for sensing acid stimuli. It is located within the sensory neurons of the peripheral nervous system and contributes to the propagation of mechanical and chemical pain signals. Intensified expression of ASIC3 correlates with peripheral tissue acidosis and inflammation,^[27]^ suggesting that it is a likely facilitator of pain transduction in the early stages of a fracture. EA has been demonstrated to alleviate inflammatory pain in rodent limbs, induced by Complete Freund’s adjuvant, by suppressing ASIC3 channel expression within the lumbar spinal ganglia, implying an analgesic effect by reducing ASIC3 channel expression.^[28]^ Sodium channels (Nav channels) are crucial for the initiation and propagation of action potentials in neurons. They have a significant role in the transmission of pain signals. Particularly during inflammatory conditions, the activity of sodium channels typically increases, leading to heightened pain sensitivity. Studies have shown that EA can reduce the expression of Nav1.7 and Nav1.8 sodium channels, which are essential for pain signal transmission in the dorsal root ganglion of mice with inflammatory pain. Additionally, EA decreases the expression of transient receptor potential vanilloid 1 (TRPV1) in dorsal root ganglion neuronal cells and the spinal cord.^[29]^ TRPV1 is a well-characterized ion channel predominantly located in small sensory nerve fibers, such as C fibers and Aδ fibers. Upon activation, calcium ions influx into nerve cells, leading to membrane depolarization and subsequent pain signaling. Additionally, there is a significant interaction between TRPV1 (transient receptor potential vanilloid 1) and Nav channels, where activation of TRPV1 enhances Nav currents.^[30]^ Studies have demonstrated that EA modulates pain-related signaling pathways by regulating the expression of TRPV1, inhibiting its excessive activation during inflammatory states, and indirectly modulating Nav currents. This multifaceted regulation contributes to its analgesic effects. Current evidence suggests that ion channels are an important mechanism for the analgesic effect of EA. However, further research is needed to address more specific issues, such as the effect of different EA parameters on ion channel activity.

### 2.3. Modulating neurotransmitters

Neurotransmitters play a crucial role in the generation, transmission, and amplification of pain signals through neurochemical pathways. The regulation of these transmitter levels may offer pain control and relief from a neurophysiological perspective.^[31]^ The analgesic effect of EA may be related to its ability to regulate the level of neurotransmitters. Recent experimental results show that the analgesic mechanism of EA has a strong correlation with the frequency parameters of EA.^[32]^

Low-frequency EA at 2 Hz has proven to be particularly effective in relieving pain caused by mechanical stimuli,^[33]^ This technique increases the levels of endorphins in the spinal cord and brain, resulting in a rise of β-endorphins in both the spinal cord and the hypothalamus. After EA treatment, there is also an increase in the mRNA levels for endorphins in the spinal cord. β-endorphins are the body’s natural pain relievers, and they work by binding to opioid receptors in the central nervous system, blocking the passage of pain signals. When endorphin receptor antagonists, such as those targeting the μ or δ receptors, are used, the pain-relieving effects of EA can be partially or completely blocked. This suggests that EA may enhance the expression and release of endogenous opioid substances like enkephalins, and this process is crucial for its pain-relief effects.^[13]^

High-frequency EA at 100 Hz raises 5-HT levels in the dorsal raphe nucleus of rats.^[15]^ Interestingly, this increase in 5-HT triggers the release of gamma-aminobutyric acid and glycine. Through interactions with 5-HT2A and 5-HT3 receptors, additional 5-HT is introduced into the spinal cord, which helps to limit pain signals carried by C fibers.^[34]^ After 100 Hz EA, there is also a significant rise in Dynorphin A levels in the cerebrospinal fluid; this peptide, derived from an indolequinone precursor, is an endogenous opioid peptide. It can bind to CB1 and CB2 cannabinoid receptors to produce pain relief, anti-inflammatory, and neuroprotective effects.^[35]^ Comparing the effects of different frequencies reveals that both influence the release of endogenous opioid peptides, but high-frequency EA might have a broader pain-relief mechanism and better analgesic effects due to enhanced 5-HT release.

A series of animal studies conducted on rabbits with ovariectomies and hysterectomies showed that the recovery period in the EA treatment group was significantly shorter compared to the control group, with superior analgesic effects.^[36]^ Similar results were observed in other animal models, including goats and horses, demonstrating significant pain relief and accelerated wound healing.^[37,38]^ Additionally, EA has shown great therapeutic potential in some case reports and clinical randomized controlled trials, particularly notable for pain relief after fracture fixation surgery (Table 1).^[39–43]^ Furthermore, EA can even alleviate persistent musculoskeletal pain in cancer patients, thereby enhancing their functional ability and quality of life.^[40]^ There were also experiments in the EA analgesia experiment did not draw a conclusion with significant differences,^[44]^ which may be due to the placebo effect. Although EA is susceptible to the placebo effect, appropriate experimental design can reduce this effect. For example, a randomized experimental group and a placebo group can be used to implement a double-blind design, using a placebo that is very similar to the actual treatment, controlling environmental factors to maintain consistency, and assessing participants expectations to understand their potential impact on the results. As an adjunct to analgesia, EA holds promise for reducing the use of analgesic drugs and minimizing systemic drug side effects.^[45]^ However, despite EA being considered a safe and effective method, a few patients in studies experienced mild side effects such as dizziness and nausea, which may affect the overall experience. Moreover, larger-scale randomized controlled trials and long-term follow-up are required to verify its effectiveness and safety.

**Table 1:** Clinical evidence of electroacupuncture analgesia

Study ID	Authors and year	Sample size	Patient characteristics	Study design	Control group	Conclusion
1	Lam Tran Quoc et al, 2022	60	patients with tibial fracture	Prospective, controlled clinical trial	Treatment using Paracetamol 1 g and Ketorolac 30 mg	EA has a significant analgesic effect within 24-48 h post-surgery.
2	Tun-Pin Hsueh et al, 2012	2	Elderly patients with closed fracture of humerus	Case report	The study did not specify a control group	EA not only plays a role in pain control, but also accelerates the healing of some fracture cases.
3	D. Del Prete et al, 2023	1	Humeral fracture patients	Case report	The study did not specify a control group	Repeated EA treatment can relieve neuropathic pain caused by brachial plexus injury.
4	Marco Di Carlo et al, 2023	1	Patients with multiple spinal fragility fractures	Case report	The study did not specify a control group	EA may be effective in complex and difficult to manage situations (such as hypersensitivity pain caused by opioids) and has potential application value.
5	Jun J et al, 2023	360	Cancer patients with chronic musculoskeletal pain	Prospective, controlled clinical trial	Conventional treatment (including analgesic drugs, physical therapy)	EA is more effective than conventional therapy in reducing chronic musculoskeletal pain in cancer survivors.

EA = electroacupuncture.

## 3. EA and inflammation regulation

After a fracture occurs, damage to blood vessels prompts interaction between blood and the surrounding tissue, triggering a chain reaction of plasma coagulation through platelet activation. This process culminates in the creation of a fibrin mesh, which expands due to various positive feedback mechanisms and eventually forms a hematoma. The hematoma plays a dual role: it controls further bleeding and serves as a focal point for inflammatory responses.^[46]^ Inflammation is a crucial initial phase in fracture healing, aiding in the removal of debris and the release of factors that promote recovery. However, an excessively intense or prolonged inflammatory response can impede the healing process. Thus, a smooth transition from inflammation to repair is essential, requiring careful regulation of both the timing and location of inflammation to ensure effective fracture healing.^[47,48]^

### 3.1. Regulating macrophage polarization

**Figure 2.**
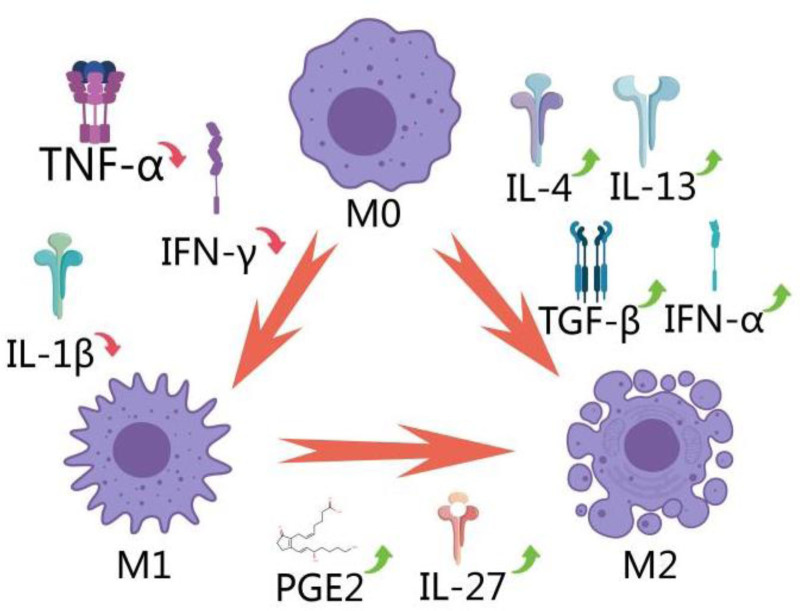
Modulation of cytokine expression and macrophage polarization by EA. EA = electroacupuncture.

In a clinical setting, it is observed that patients with brain injuries and fractures experience a higher healing rate of fractures compared to patients with simple fractures.^[49,50]^ This is attributed to the mechanism where brain injury promotes fracture healing through the sympathetic nervous system’s β-adrenergic receptor signaling pathway. This process involves an increase in bone marrow hematopoietic stem cells and their differentiation into M2 macrophages, thereby accelerating the transition from M1 to M2 macrophages. M1 macrophages, or “classically activated” macrophages, play a crucial role in inflammation by helping clear infections and triggering an immune response. On the other hand, M2 macrophages, known as “alternatively activated” macrophages, help reduce inflammation and support tissue repair and healing.^[51]^ This rapid shift in macrophage polarization is crucial in the early stages of fracture healing as it reduces inflammation and promotes recovery.^[52]^ However, exploiting brain injuries to improve fracture healing is not practical. Research indicates that EA may provide similar therapeutic benefits (Fig. 2).

In an animal experiment on skeletal muscle inflammation, EA significantly reduced the expression of M1 macrophage markers (such as CD86) and increased the expression of M2 macrophage markers (such as CD163).^[53]^ In another study on skeletal muscle contusion, it was found that EA could lower the level of interferon gamma while increasing interleukin-4 (IL-4), interleukin-13 (IL-13), and interferon alpha, thereby promoting the polarization shift from M1 to M2 macrophages.^[16]^ Moreover, in a mouse model of acute colitis induced by dextran sulfate sodium, EA treatment led to an increase in the M2/M1 macrophage ratio, effectively reducing acute colonic inflammation.^[54]^ Additionally, in a rat model of spinal cord injury, EA reduced M1 macrophages and increased M2 macrophages, thereby inhibiting the inflammatory response.^[55]^ These experiments demonstrate that EA has significant therapeutic potential in inflammation regulation, particularly in the regulation of macrophage polarization. Regrettably, there is currently no definitive evidence proving the effectiveness of EA in clinical regulation of macrophage polarization types, which requires further research.

It is important to note that EA does not uniformly affect the M1 to M2 macrophage polarization shift and may sometimes have the opposite effect.For example, in mouse experiments involving surgically induced stress, the stress response encouraged a shift towards M2 macrophages, which impaired immune function. EA has been observed to inhibit stress responses in these conditions.Compared to mice under surgical stress, those treated with EA showed lower cortisol levels in peripheral blood and increased expression of cytokines such as Inducible Nitric Oxide Synthase, Tumour Necrosis Factor-alpha (TNF-α), and interleukin-1 beta (IL-1β).This response leads to an increase in M1-related factors and a decrease in M2-related factors.^[56]^This phenomenon of dual regulation indicates that the effect of EA is closely related to the body’s state, and is not a point-to-point precise control. A more accurate description is that EA regulates the body in a multifaceted, macro-level manner, tending to restore the body’s abnormal state to a normal state.

### 3.2. Regulating inflammatory cytokines

Beyond merely modulating macrophage polarization, EA has a broader regulatory effect on inflammation by influencing the expression of specific cytokines and inflammatory pathways.^[45]^ In a rat model of femoral fracture surgery, postoperative EA treatment significantly lowered the serum concentrations of IL-1β, IL-6, and TNF-α, thereby effectively mitigating the postoperative peripheral inflammatory response.^[57]^ Furthermore, EA can curtail myeloperoxidase activity within tissues^[58]^ and temper oxidative stress responses by reducing the expression of molecules such as malondialdehyde, Cyclooxygenase-two, Prostaglandin E, and Nitric Oxide Synthase.^[14]^ As a result, tissue damage and inflammation are diminished. EA also possesses the ability inhibits the activity of NF-κB, neutralizing the enhancing effect of the NF-κB signaling pathway on pro-inflammatory cytokines such as TNF-α and IL-1β.^[59]^ In clinical studies, EA also exhibits excellent anti-inflammatory effects. Dennis Grech and others conducted low-frequency EA on 20 patients under general anesthesia to explore its impact on postoperative recovery. To avoid the placebo effect, the study was conducted on anesthetized patients using a prospective double-blind randomized trial design. The results showed that patients receiving EA required 60% less analgesic medication postoperatively. Furthermore, EA significantly enhanced the production of Transforming Growth Factor β1 (TGF-β1).^[60]^ In another clinical study on the effects of EA on inflammation after cranial surgery, 44 patients participated and were randomly divided into the EA group and the control group. The EA group received treatment 6 times within 8 days post-surgery, while the control group received only standard postoperative care. The results showed that the levels of IL-1β and TNF-α on days 2 and 7 post-surgery were significantly lower in the EA group than in the control group.^[61]^ These studies provide clinical evidence for the anti-inflammatory effects of EA, but the sample size is small, necessitating larger-scale clinical trials in the future to confirm its mechanisms and efficacy.

## 4. Role of EA in tissue repair

The positive effects of EA on tissue healing are mainly reflected in 2 aspects: first, EA can improve local blood supply to fractures, including promoting neovascularization and enhancing blood circulation, which is achieved by stimulating the production and release of VEGF, as well as increasing the expression of some important mRNA and exosomal miR-210 and other cytokines. On the other hand, EA can promote bone repair, including inducing stem cell differentiation and regulating the expression levels of bone morphogenetic protein-two (BMP-2).

### 4.1. EA can improve the local blood supply of fracture

A fracture typically leads to localized vascular damage, resulting in the formation of a hematoma. This is a crucial process for supplying the nutrients and oxygen necessary for cell growth and metabolism.^[62]^ Prominent growth factors, such as VEGF, are released by endothelial cells and activated platelets, promoting angiogenesis.^[63,64]^ The nascent blood vessels formed through this process enable mesenchymal stem cells from nearby tissues, like the periosteum, to migrate to the fracture site, thereby accelerating the healing process.^[65,66]^ Therefore, angiogenesis is vital for fracture healing. In a experiment involving a rat model with cerebral ischemia-reperfusion injury, EA treatment stimulated an increase in Wnt3a and β-catenin. This led to the activation of the Wnt/β-catenin pathway, enhancing the expression of angiogenic factors, including VEGF, Ang2, and stromal cell-derived factor. Notably, the upregulation of these factors is linked to improved new blood vessel formation.^[67]^

### 4.2. EA’s impact on mRNA levels in angiogenesis

In a rat model of Middle Cerebral Artery Occlusion (MCAO), the application of 15 Hz low-frequency EA significantly increased EphB4 and EphrinB2 mRNA levels in the ischemic region. EphB4 activates Src family kinases, and EphrinB2 promotes Phosphatidylinositol 3-kinase (PI3K) activation, leading to increased VEGF expression. This boost in VEGF results in endothelial cell proliferation and the creation of new blood vessels.^[17]^ Additionally, EA enhances cerebral tissue erythropoietin, triggering the downstream Src phosphorylation signaling pathway, further boosting VEGF expression.^[68]^ This increase in VEGF raises Notch ligand expression, activating the Notch signaling pathway.^[69]^ The Notch pathway, through integrating spatial and temporal signals provided by VEGF, regulates endothelial cell differentiation and vasculogenesis.^[70]^

### 4.3. EA and exosomal miR-210 in angiogenesis

Research has shown that post-EA at the “Ren Zhong” (DU26) and “Bai Hui” (DU20) points in MCAO mice results in the separation of EA-derived exosomes (EA-EXOs) from the serum, which contain increased levels of miR-210. This suggests that EA may enhance the expression of exosomal miR-210 induced by MCAO, thus promoting angiogenesis.^[71]^ Further analysis indicated that introducing EA-EXOs into hypoxia-stimulated human umbilical vein endothelial cell cultures increased miR-210 expression. EA-EXOs, along with miR-210 mimics, significantly raised the expression of hypoxia-inducible factor 1α, VEGF, and Notch 1 proteins and mRNA in hypoxic human umbilical vein endothelial cells, enhancing their proangiogenic effect.^[72]^

### 4.4. EA’s influence on blood flow and angiogenesis

**Figure 3.**
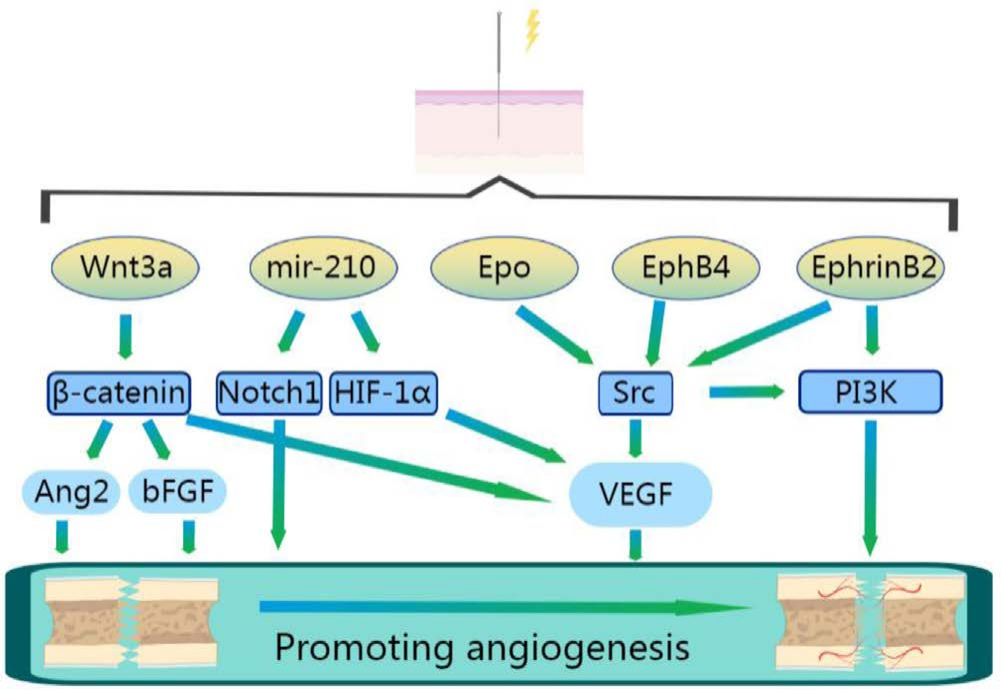
Mechanisms of EA in promoting angiogenesis. EA = electroacupuncture.

EA enhances angiogenesis through various pathways (Fig. 3),and simultaneously boosts local blood flow velocity. A study on cutaneous microcirculation in healthy individuals found that low-frequency EA significantly improved both microcirculation and cardiac rate, while high-frequency EA reduced microcirculation without affecting heart rate.^[73]^ Complementary research on direct current stimulation of the lower extremities showed that EA increased blood flow velocity near observation points, maintained for 5 minutes after stimulus removal.^[74]^ In a rat model of cerebral ischemia, moderate EA stimulation (intensity of approximately 1.0 mA and frequency of 5–20 Hz) optimally increased cerebral blood flow, achieving a peak amplitude near 100%. Meanwhile, stimulation intensities below 0.6 mA or frequencies above 40 Hz showed no effect on increasing cerebral blood flow.^[75]^

### 4.5. EA can promote bone repair

Following a fracture, the formation of a hematoma due to ruptured local blood vessels serves as an initial response site, promoting inflammatory reactions and providing essential mechanical stability.^[76]^ Once the acute inflammatory phase subsides, the repair stage begins, culminating in the development of a callus composed of bone and cartilage at the fracture site. This callus offers superior mechanical support compared to the initial hematoma and plays a critical role in fracture healing.^[77]^ The presence of chondrocytes is key in this process, as a deficiency or reduction can delay healing. Evidence indicates that EA can accelerate fracture healing.^[78]^ In a study with a rat model of tibial fractures, 50 Hz pulsed EA applied to the fracture area yielded improved results in bone formation, bone mineral density, and fracture strength compared to sham and control groups.^[79]^ Moreover, EA has shown favorable therapeutic outcomes in clinical studies. In clinical research involving 50 patients after high tibial osteotomy and 600 patients with fractures of the middle and lower third of the tibia and fibula, the EA group demonstrated notable efficacy in shortening clinical healing time and reducing the incidence of delayed healing.^[11,12]^ However, in the study by Lam et al, although EA had excellent analgesic effects, there was no significant difference between the EA group and the control group in fracture healing. The reason for this conclusion may be the excessively high frequency of EA, as most experiments that concluded EA promotes bone repair focused on frequency parameters between 2HZ and 15HZ.

### 4.6. EA induces stem cell differentiation

EA-assisted tissue healing involves the production of various cytokines. Research suggests that low-frequency EA can induce the differentiation of rat bone marrow mesenchymal stem cells into chondrocytes by upregulating Sox9 mRNA expression. This encourages chondrocyte migration to fracture sites, thus promoting healing.^[80]^ In tests where rat fibula fractures were treated with EA, there was an increase in BMP-2 expression, as shown by immunohistochemical staining. The group receiving 50 Hz EA treatment exhibited a higher cell count and faster fracture healing than the control group, as confirmed by histological and radiological evaluations.^[81]^

### 4.7. EA increased BMP-2 expression

BMP-2 is a multifunctional growth factor critical in the early stages of fracture healing; it regulates the process of osteogenesis by promoting the proliferation, differentiation, and apoptosis of osteoprogenitor cells. In the early phase of fracture healing, BMP-2 attracts osteoprogenitor cells to the injury site, facilitating cartilage and new bone formation. Activation of the BMP signaling pathway is crucial for fracture healing because it not only promotes the differentiation of osteoblasts but also regulates angiogenesis and cell recruitment. However, in an inflammatory environment, BMP signaling may be inhibited, leading to insufficient osteogenesis.^[82–84]^Some experiments on EA suggest that EA can increase BMP-2 expression, which may be one of the mechanisms by which EA promotes bone repair. For example, in a rabbit intervertebral disc degeneration model, EA with alternating frequencies of 2 to 15 Hz increased BMP-2 expression in the disc tissue.^[85]^ Similarly, in a streptozotocin-induced diabetic mouse model, high-frequency EA (100 Hz) enhanced BMP-2 expression in the gut.^[86]^ Collectively, these findings suggest a direct positive correlation between EA stimulation and increased BMP-2 expression, highlighting EA’s regulatory impact on BMP-2 as a potential mechanism for promoting tissue healing and bone formation.

## 5. Discussion

EA, employed as an auxiliary treatment method, positively impacts pain alleviation, anti-inflammatory responses, vascular regeneration, and tissue formation throughout the fracture healing course. Conventional pain-relief strategies, such as opioid and non-steroidal anti-inflammatory drugs usage, may increase the risk of nonunion fractures or addiction.^[87,88]^ Conversely, EA does not increase these risks. When combined with systemic analgesics, it can exert a better analgesic effect, thereby reducing the use of analgesic drugs.^[89]^ In animal studies, combining EA with a low dose of indomethacin (INDO) has been shown to enhance pain relief and potentially reduce the need for medication.^[90]^ Later, In a clinical trial exploring EA as an adjunct therapy for patients with knee osteoarthritis, the combination of EA and etoricoxib proved significantly more effective in relieving pain than both sham EA with drug therapy and drug therapy alone.^[91]^ This combined synergistic effect may be a new way to reduce the side effects of analgesic drugs. Additionally, owing to its wide-reaching analgesic range, EA is applicable to various forms of pain relief, including musculoskeletal and neuropathic pains.^[38]^ Such benefits qualify EA as particularly valuable for early post-fracture pain management and positively influence subsequent healing procedures. In addition, through the modulation of macrophage polarization types and inflammatory cytokine expression, EA accelerates the healing transition from the inflammatory stage to the repair phase, considerably reducing the duration of fracture healing. This factor has substantial implications for managing medical costs and reducing the complications arising from extended immobilization.

Recent studies exploring EA have demonstrated its ability to bidirectionally modulate the heart rate and blood flow velocity, thereby facilitating the rectification of pathological states. Although this regulatory effect may diminish over time, it emphasizes the clinical safety of EA. In terms of parameter design, EA frequency plays a pivotal role in interpreting its curative effects, likely rooted in the distinctive physiology of the human body. Considering that various tissues exhibit different bioelectric conduction frequencies,^[92]^ EA application at diverse frequencies could yield a range of facilitative or suppressive results in electrical conduction. Notably, EA uniquely contributes to tissue repair, as evidenced by its modulation of cell differentiation, healing-associated pathways, and cytokine expression. Specifically, when the healing processes are compromised, EA could potentially augment these impaired mechanisms, highlighting its therapeutic potential in reducing the risk of delayed healing in patients with underlying medical conditions.

EA, a beneficial adjuvant therapy for fracture healing, is effective and safe as an interventional therapy. However, this study had certain limitations. Primarily, the effectiveness of EA is significantly influenced by stimulation location, with most experimental results originating from specific points. For example, changes in macrophage polarization have been observed after stimulation at acupoints such as ST36,^[54]^ GV9,^[54]^ and ST25,^[93]^ while an increase in the expression of VEGF has been recorded at acupoints GV26^[68]^ and GV14.^[17]^ Furthermore, EA parameters can substantially influence treatment outcomes. This phenomenon is demonstrated in a rat model of cerebral ischemia where frequencies and intensities within 5 to 20 Hz and exceeding 0.6mA were found to increase cerebral blood flow.^[75]^ The analgesic mechanisms also exhibit variations at different frequencies, notably at lower (2 Hz) and higher (100 Hz) frequencies. Thus, it can be deduced that the site of EA stimulation primarily governs the therapeutic effect, while EA parameters serve as pivotal determinants in either enhancing or undermining the response.

Current research indicates that EA possesses attributes that mitigate the side effects commonly associated with mainstream therapeutic modalities. It achieves this by modulating the expression of cytokines and facilitating shifts in cellular polarization that favor fracture healing. This suggests that integrating EA into clinical practice could substantially reduce the risk of delayed union and nonunion of fractures, without significantly increasing healthcare costs. Future research should, therefore, focus on optimizing the use of these benefits to enhance clinical outcomes efficiently.

## 6. Future directions

In recent years, EA has shown potential in promoting fracture healing and shortening recovery time. However, several issues still need to be addressed: The molecular mechanisms by which EA enhances fracture healing are not yet fully understood, especially the effects of electrical stimulation on the proliferation and differentiation of bone cells and its role in regulating inflammatory responses and promoting angiogenesis. Although EA has demonstrated its unique effects in some clinical studies, larger scale and more rigorous randomized controlled trials are still required to ensure its safety and efficacy, and to summarize the therapeutic patterns of EA, to avoid potential side effects in different populations. The nature of the interaction during the initial phase between the electrical field induced by EA and the bioelectric field at acupuncture sites remains unclear. Understanding the mechanism of EA is crucial by studying the electrical properties of tissues and clarifying the significance of differences in these properties. Some diseases may increase the risk of fractures and hinder fracture healing; this is particularly challenging in patients with comorbidities such as osteoporosis or diabetes. Investigating the long-term effects of EA on bone regeneration in difficult situations is of great significance.

## Author contributions

**Conceptualization:** Yu-Jun Gao.

**Data curation:** Yin-cang Wang, Qing Wen.

**Investigation:** De-lai Zhao, Hao-Xin Shi.

**Writing – original draft:** Yu-Jun Gao.

**Writing – review & editing:** Yu-Jun Gao, Shu-Ren Wang.
